# The genome sequence of a longhorn beetle, *Rhagium mordax *(Degeer, 1775)

**DOI:** 10.12688/wellcomeopenres.23668.1

**Published:** 2025-02-07

**Authors:** Maxwell V. L. Barclay, Dmitry Telnov, Natural History Museum Genome Acquisition Lab, Darwin Tree of Life Barcoding collective, Wellcome Sanger Institute Tree of Life Management, Samples and Laboratory team, Wellcome Sanger Institute Scientific Operations: Sequencing Operations, Wellcome Sanger Institute Tree of Life Core Informatics team, Tree of Life Core Informatics collective, Darwin Tree of Life Consortium

**Affiliations:** 1 Natural History Museum, London, England, UK; 2 Daugavpils University, Daugavpils, Latvia; 3 University of Latvia, Rīga, Latvia

**Keywords:** Rhagium mordax, longhorn beetle, genome sequence, chromosomal, Coleoptera

## Abstract

We present a genome assembly from an individual female specimen of *Rhagium mordax* (longhorn beetle; Arthropoda; Insecta; Coleoptera; Cerambycidae). The genome sequence has a total length of 775.60 megabases. Most of the assembly (99.53%) is scaffolded into 10 chromosomal pseudomolecules. The mitochondrial genome has also been assembled and is 16.68 kilobases in length. Gene annotation of this assembly on Ensembl identified 11,937 protein-coding genes.

## Species taxonomy

Eukaryota; Opisthokonta; Metazoa; Eumetazoa; Bilateria; Protostomia; Ecdysozoa; Panarthropoda; Arthropoda; Mandibulata; Pancrustacea; Hexapoda; Insecta; Dicondylia; Pterygota; Neoptera; Endopterygota; Coleoptera; Polyphaga; Cucujiformia; Chrysomeloidea; Cerambycidae; Lepturinae; Rhagiini; *Rhagium*; *Rhagium mordax* (Degeer, 1775) (NCBI:txid295679)

## Background

The Cerambycidae Latreille, 1802, commonly known as longhorned beetles, longicorns, capricorns, round-headed borers, timber beetles, or sawyer beetles, comprises one of the largest families of the order Coleoptera with at least 35,000 species worldwide ( Rossa & Goczał, 2021). Moreover, cerambycids represents one of most diverse, ecologically, and economically important group of beetles. The family is of cosmopolitan distribution, most speciose in the tropical regions. Longhorn beetle larvae are usually saproxylic, xylophagous or herbicolous, feed on decaying or live plant tissues and provide essential ecosystem service by rapid recycling dead wood into humus. Adults are phytophagous, feeding on green or withered plants, many species are anthophilous or forage for fermented tree sap. The English names of the family refer to the elongate antennae of these beetles or to their connection with wood. Nine extant subfamilies are recognized within Cerambycidae ( Bouchard *et al.*, 2011). In the British fauna, there are 68 established species of longhorn beetles known presently ( Duff, 2018). The genus *Rhagium* Fabricius, 1775 is represented in the Palaearctic Region by three subgenera and 26 species and subspecies ( Danilevsky, 2020). Seven species and subspecies of *Rhagium* occur in Europe ( Danilevsky, 2020), three of which are present in the British fauna ( Duff, 2020).

*Rhagium mordax* (DeGeer, 1775) is placed in the subfamily Lepturinae tribe Rhagiini ( Danilevsky, 2020). The species is assigned to the subgenus *Megarhagium* Reitter, 1913. Adults of the species are well-defined morphologically and resemble only those of *R. (M.) sycophanta* (Schrank, 1781), a slightly larger congener which does not occur in Britain (although erroneously listed as British in Danilevsky, 2020). Paulus (1969) provided a key to the Central European *Rhagium* larvae. It is hoped that genomic data may help clarify the identification of immature *Rhagium*.

*Rhagium mordax* is a trans-Palaearctic species widely distributed from northern and eastern Spain and the British Isles towards the eastern Siberia and Kazakhstan ( Danilevsky, 2020 and references therein; GBIF Secretariat, 2024). Danilevsky (2020) list the species from 37 European countries, including the UK. Generally, it is much less abundant in southern and southeastern Europe than in the zone of temperate and boreal forests. In Asia, the species is distributed in western and eastern Siberia, northern Kazakhstan, with an isolated subpopulation in Turkmenistan ( Danilevsky, 2020 and references therein). The European extent of occurrence and area of occupancy of this species are both strongly above the thresholds for a threatened species ( Dodelin *et al.*, 2017).

*Rhagium mordax* is a forest species. Its larvae are saproxylic and polyphagous, developing for 2–3 years between bark and hardwood of decaying *Alnus* spp., *Betula* spp., *Fagus sylvatica*, *Populus tremula*, *Quercus* spp., occasionally also coniferous trees ( Duff, 2016; Koch, 1992; Nikitsky *et al.*, 1996; Palm, 1959). Larval development takes two years in the northern and eastern parts of the range, pupae are found throughout the year ( Nikitsky *et al.*, 1996; Palm, 1959). This species uses wood in a broad range of conditions of for larval development, including snags and fallen trunks in shady or sun exposed, wet or moderately dry conditions. Although generally associated with deciduous trees, live larvae have been collected under bark of pine *Pinus* sp. washed up on a beach in North Devon (M.V.L. Barclay personal observation). The species is described as eurytopic, silvicol, corticol, lignicol, xylophagous and anthophilous ( Koch, 1992). Adult beetles occur in forests, on forest edges, hedgerows and clearing. They feed on pollen of various blossoming plants, in Britain most often the blossom of hawthorn *Crataegus* (Rosaceae); they have also been recorded feeding on fermented tree sap (D. Telnov, personal observations in Latvia).

The sequenced larva was sampled in April from under bark of a dead and decaying oak trunk *Quercus robur*. In Britain, adults have been found all year ( Duff, 2016), but mostly inside the larval substrate, becoming active outside the wood in Spring and early Summer. In the northern and eastern parts of the distribution area, the adults appear in July to August.

*Rhagium mordax* is a common and widespread species in United Kingdom and recorded in England, Wales and Scotland northwards to West Sutherland as well as in Ireland ( Duff, 2016). The species was not listed in the national Red Data Book ( Shirt, 1987), the present status of the species in the United Kingdom is Least Concern (LC) ( Alexander, 2014).

The specimen used for sequencing, a mature larva under oak bark at Bookham Common, Surrey, southern England, was collected and identified by M.V.L. Barclay.

## Genome sequence report

### Sequencing data

**Figure 1.**
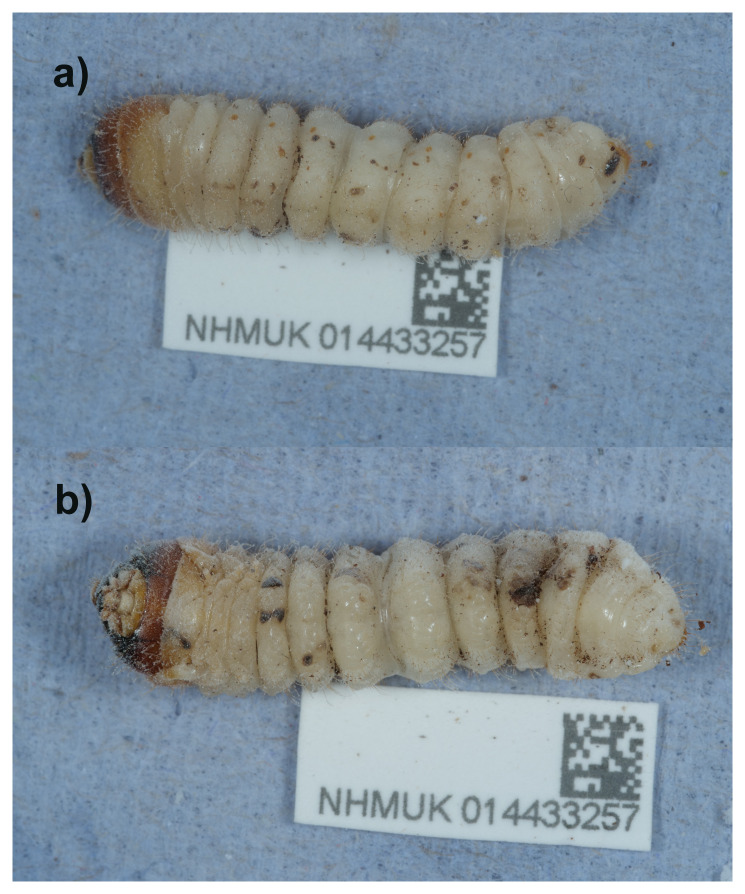
Photographs of the *Rhagium mordax* (icRhaMord1) specimen used for genome sequencing.

The genome of *Rhagium mordax* ( Figure 1) was sequenced using Pacific Biosciences single-molecule HiFi long reads, generating a total of 21.80 Gb (gigabases) from 2.31 million reads, providing an estimated 26-fold coverage. Hi-C sequencing produced 94.64 Gb from 626.77 million reads. Specimen and sequencing details are summarised in Table 1.

**Table 1.** Specimen and sequencing data for *Rhagium mordax*.

Project information			
**Study title**	*Rhagium mordax*		
**Umbrella BioProject**	PRJEB63422		
**BioSample**	SAMEA111458823		
**NCBI taxonomy ID**	295679		
Specimen information			
**Technology**	**ToLID**	**BioSample accession**	**Organism part**
**PacBio long read sequencing**	icRhaMord1	SAMEA111458864	Whole organism
**Hi-C sequencing**	icRhaMord1	SAMEA111458823	Whole organism
Sequencing information			
**Platform**	**Run accession**	**Read count**	**Base count (Gb)**
**Hi-C Illumina NovaSeq 6000**	ERR11606307	6.27e+08	94.64
**PacBio Sequel IIe**	ERR11593795	2.31e+06	21.8

### Assembly statistics

The primary haplotype was assembled, and contigs corresponding to an alternate haplotype were also deposited in INSDC databases. The assembly was improved by manual curation, which corrected 197 misjoins or missing joins and removed 47 haplotypic duplications. These interventions reduced the total assembly length by 1.82%, decreased the scaffold count by 47.73%, and increased the scaffold N50 by 0.56%. The final assembly has a total length of 775.64 Mb in 68 scaffolds, with 446 gaps, and a scaffold N50 of 80.11 Mb ( Table 2).

**Table 2.** Genome assembly data for *Rhagium mordax*, icRhaMord1.1.

Genome assembly		
Assembly name	icRhaMord1.1	
Assembly accession	GCA_963680705.1	
*Accession of alternate haplotype*	*GCA_963680695.1*	
Span (Mb)	775.60	
Number of contigs	515	
Number of scaffolds	68	
Longest scaffold (Mb)	134.33	
Assembly metrics *		*Benchmark*
Contig N50 length (Mb)	2.9	*≥ 1 Mb*
Scaffold N50 length (Mb)	80.1	*= chromosome N50*
Consensus quality (QV)	Primary: 61.2; alternate: 60.6; combined 60.9	*≥ 40*
*k*-mer completeness	Primary: 86.98%; alternate: 72.20%; combined: 97.79%	*≥ 95%*
BUSCO v5.4.3 lineage: endopterygota_odb10	C:99.0%[S:97.8%,D:1.2%], F:0.6%,M:0.4%,n:2,124	*S > 90%, D < 5%*
Percentage of assembly mapped to chromosomes	99.53%	*≥ 90%*
Sex chromosomes	Not identified	*localised homologous pairs*
Organelles	Mitochondrial genome: 16.68 kb	*complete single alleles*
Genome annotation of assembly GCA_963680705.1 at Ensembl		
Number of protein-coding genes	11,937	
Number of non-coding genes	1,505	
Number of gene transcripts	19,101	

* Assembly metric benchmarks are adapted from Rhie *et al.* (2021) and the Earth BioGenome Project Report on Assembly Standards September 2024. ** BUSCO scores based on the endopterygota_odb10 BUSCO set using version 5.4.3. C = complete [S = single copy, D = duplicated], F = fragmented, M = missing, n = number of orthologues in comparison. A full set of BUSCO scores is available at https://blobtoolkit.genomehubs.org/view/Rhagium_mordax/dataset/GCA_963680705.1/busco.

**Figure 2.**
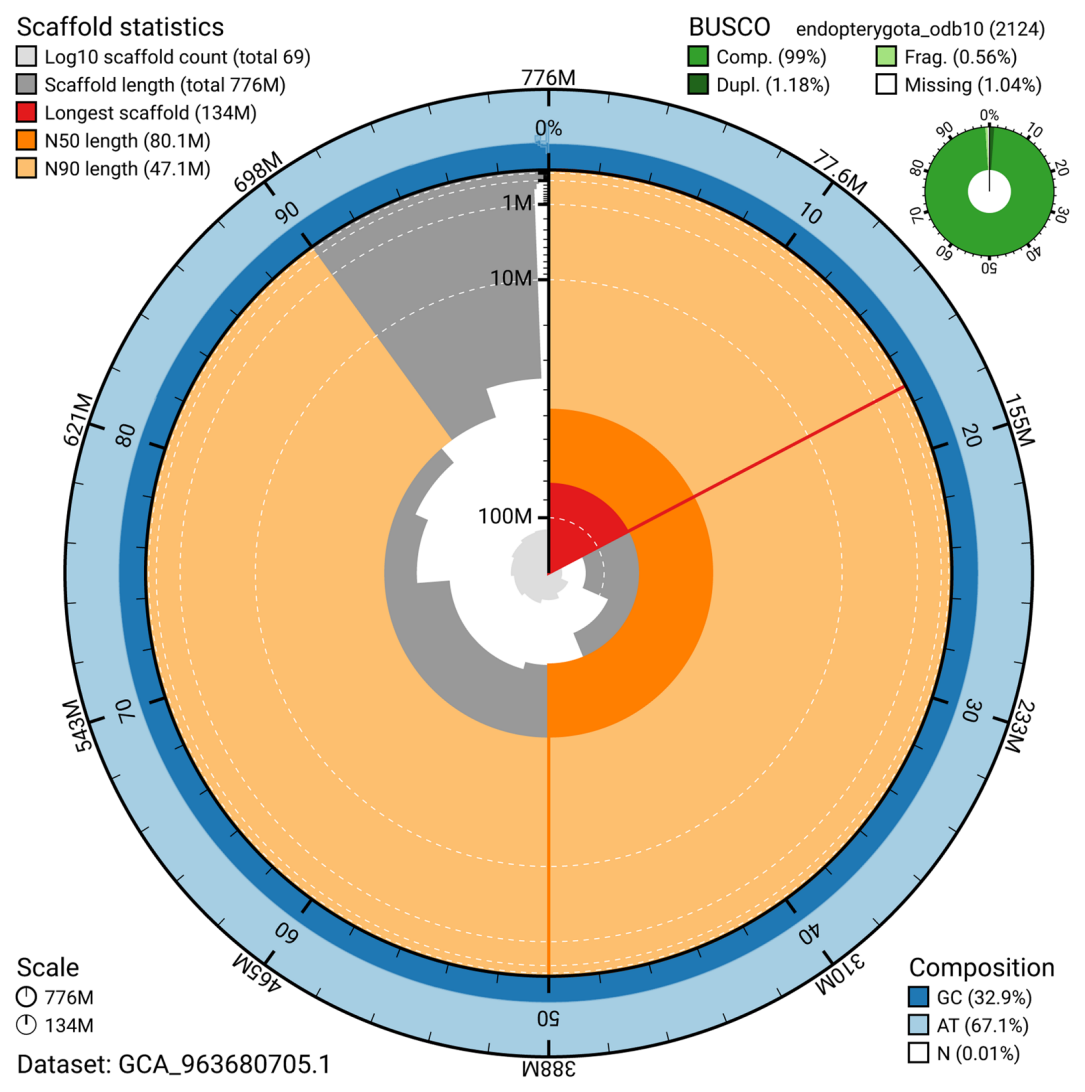
Genome assembly of *Rhagium mordax*, icRhaMord1.1: metrics. The BlobToolKit snail plot provides an overview of assembly metrics and BUSCO gene completeness. The circumference represents the length of the whole genome sequence, and the main plot is divided into 1,000 bins around the circumference. The outermost blue tracks display the distribution of GC, AT, and N percentages across the bins. Scaffolds are arranged clockwise from longest to shortest and are depicted in dark grey. The longest scaffold is indicated by the red arc, and the deeper orange and pale orange arcs represent the N50 and N90 lengths. A light grey spiral at the centre shows the cumulative scaffold count on a logarithmic scale. A summary of complete, fragmented, duplicated, and missing BUSCO genes in the endopterygota_odb10 set is presented at the top right. An interactive version of this figure is available at https://blobtoolkit.genomehubs.org/view/GCA_963680705.1/dataset/GCA_963680705.1/snail.

**Figure 3.**
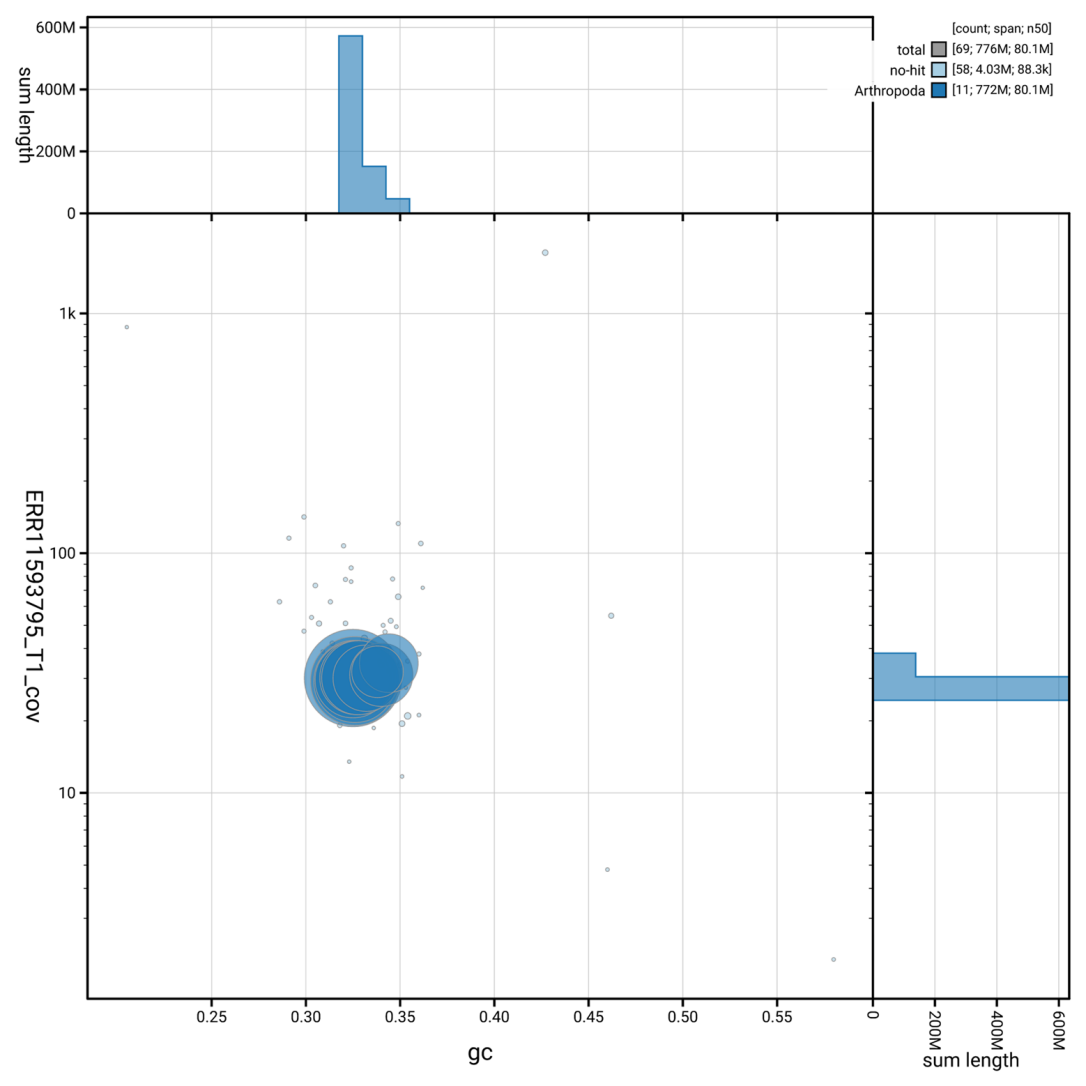
Genome assembly of *Rhagium mordax*, icRhaMord1.1: BlobToolKit GC-coverage plot showing sequence coverage (vertical axis) and GC content (horizontal axis). The circles represent scaffolds, with the size proportional to scaffold length and the colour representing phylum membership. The histograms along the axes display the total length of sequences distributed across different levels of coverage and GC content. An interactive version of this figure is available at https://blobtoolkit.genomehubs.org/view/GCA_963680705.1/dataset/GCA_963680705.1/blob.

**Figure 4.**
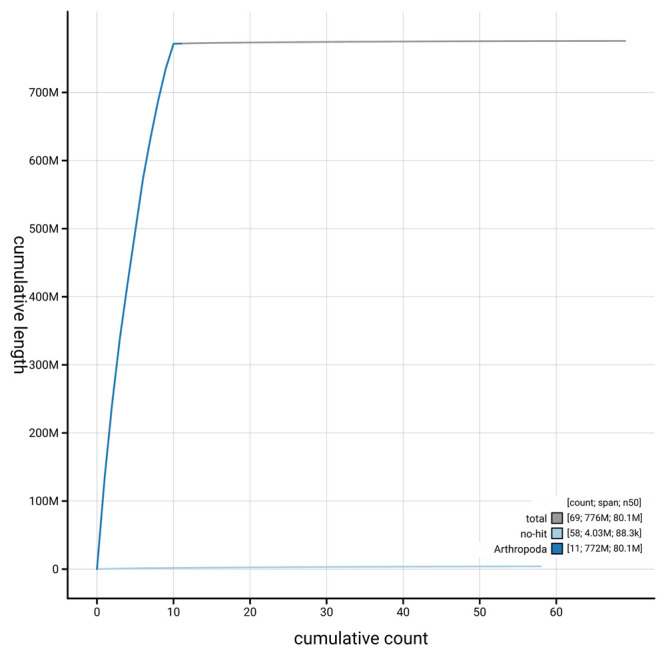
Genome assembly of *Rhagium mordax* icRhaMord1.1: BlobToolKit cumulative sequence plot. The grey line shows cumulative length for all scaffolds. Coloured lines show cumulative lengths of scaffolds assigned to each phylum using the buscogenes taxrule. An interactive version of this figure is available at https://blobtoolkit.genomehubs.org/view/GCA_963680705.1/dataset/GCA_963680705.1/cumulative.

The snail plot in Figure 2 provides a summary of the assembly statistics, indicating the distribution of scaffold lengths and other assembly metrics. Figure 3 shows the distribution of scaffolds by GC proportion and coverage. Figure 4 presents a cumulative assembly plot, with separate curves representing different scaffold subsets assigned to various phyla, illustrating the completeness of the assembly.

**Figure 5.**
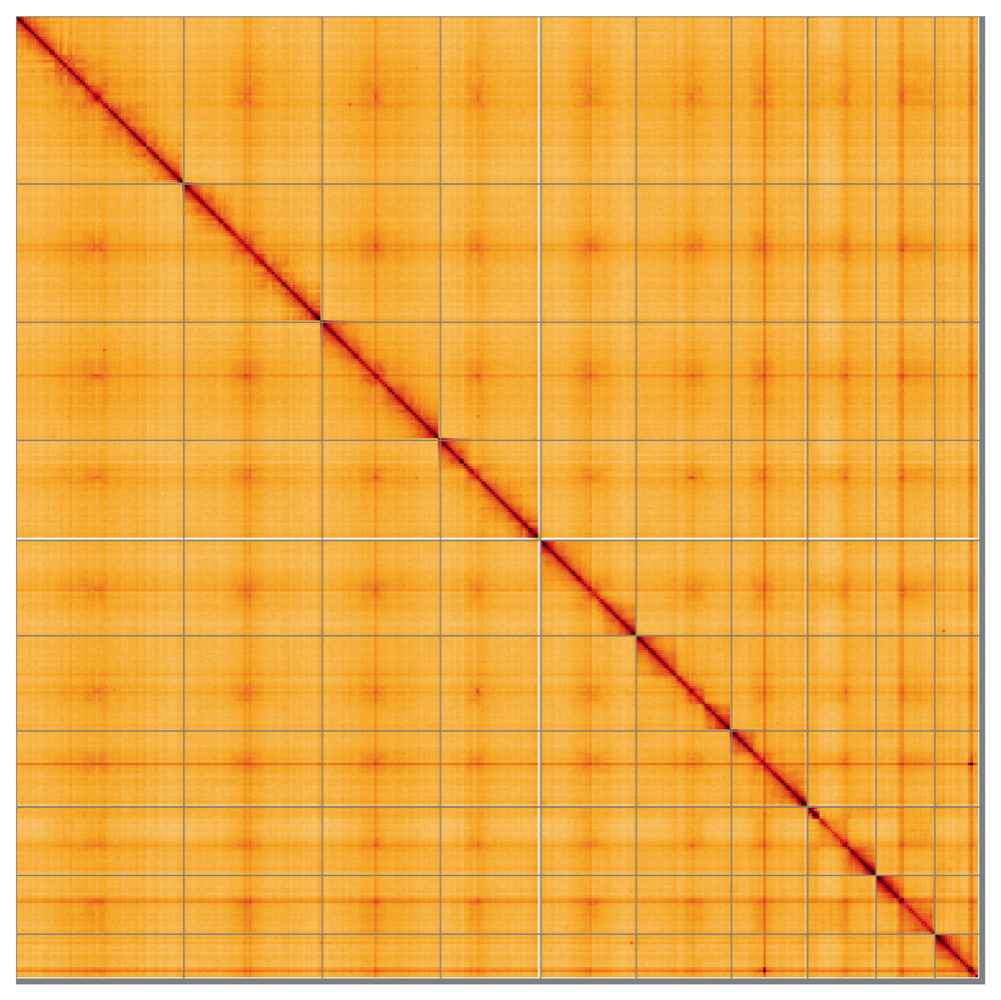
Genome assembly of *Rhagium mordax* icRhaMord1.1: Hi-C contact map of the icRhaMord1.1 assembly, visualised using HiGlass. Chromosomes are shown in order of size from left to right and top to bottom. An interactive version of this figure may be viewed at https://genome-note-higlass.tol.sanger.ac.uk/l/?d=YRLlDEwzQjiwvrfgTh3rVA.

Most of the assembly sequence (99.47%) was assigned to 10 chromosomal-level scaffolds. These chromosome-level scaffolds, confirmed by Hi-C data, are named according to size ( Figure 5; Table 3). During curation, it was noted that the sample is homogametic sex (female) but X chromosome could not be identified.

**Table 3.** Chromosomal pseudomolecules in the genome assembly of *Rhagium mordax*, icRhaMord1.

INSDC accession	Name	Length (Mb)	GC%
OY796656.1	1	134.33	32.5
OY796657.1	2	110.84	32.5
OY796658.1	3	94.63	32.5
OY796659.1	4	80.11	32.5
OY796660.1	5	76.39	32.5
OY796661.1	6	76.33	33.0
OY796662.1	7	60.86	33.0
OY796663.1	8	54.87	34.0
OY796664.1	9	47.15	34.5
OY796665.1	10	35.99	34.0
OY796666.1	MT	0.02	20.5

The mitochondrial genome was also assembled. This sequence is included as a contig in the multifasta file of the genome submission, and as a separate fasta file with accession OY796666.1.

### Assembly quality metrics

The estimated Quality Value (QV) and *k*-mer completeness metrics, along with BUSCO completeness scores, were calculated for each haplotype and the combined assembly. The QV reflects the base-level accuracy of the assembly, while *k*-mer completeness indicates the proportion of expected *k*-mers identified in the assembly. BUSCO scores provide a measure of completeness based on benchmarking universal single-copy orthologues.

The primary haplotype has a QV of 61.2, and the combined primary and alternate assemblies achieve an estimated QV of 60.9. The *k*-mer completeness for the primary haplotype is 86.98%, and for the alternate haplotype it is 72.20%. The combined primary and alternate assemblies achieve a *k*-mer completeness of 97.79%. BUSCO analysis using the endopterygota_odb10 reference set ( *n* = 2,124) indicated a completeness score of 99.0% (single = 97.8%, duplicated = 1.2%).

Table 2 provides assembly metric benchmarks adapted from Rhie *et al.* (2021) and the Earth BioGenome Project Report on Assembly Standards September 2024. The assembly achieves the EBP reference standard of 6.C.61.

### Genome annotation report

The *Rhagium mordax* genome assembly (GCA_963680705.1) was annotated at the European Bioinformatics Institute (EBI) on Ensembl Rapid Release. The resulting annotation includes 19,101 transcribed mRNAs from 11,937 protein-coding and 1,505 non-coding genes ( Table 2; https://rapid.ensembl.org/Rhagium_mordax_GCA_963680705.1/Info/Index). The average transcript length is 12,324.32. There are 1.42 coding transcripts per gene and 5.16 exons per transcript.

## Methods

### Sample acquisition and DNA barcoding

A female larval specimen of *Rhagium mordax* (specimen ID NHMUK014433257, ToLID icRhaMord1) was collected from Bookham Common, England, United Kingdom (latitude 51.29, longitude –0.39) on 2021-04-18. The specimen was collected and identified by Maxwell Barclay (Natural History Museum) and preserved by dry freezing at –80 °C.

The initial identification was verified by an additional DNA barcoding process according to the framework developed by Twyford *et al.* (2024). A small sample was dissected from the specimens and stored in ethanol, while the remaining parts were shipped on dry ice to the Wellcome Sanger Institute (WSI). The tissue was lysed, the COI marker region was amplified by PCR, and amplicons were sequenced and compared to the BOLD database, confirming the species identification ( Crowley *et al.*, 2023). Following whole genome sequence generation, the relevant DNA barcode region was also used alongside the initial barcoding data for sample tracking at the WSI ( Twyford *et al.*, 2024). The standard operating procedures for Darwin Tree of Life barcoding have been deposited on protocols.io ( Beasley *et al.*, 2023).

### Nucleic acid extraction

The workflow for high molecular weight (HMW) DNA extraction at the Wellcome Sanger Institute (WSI) Tree of Life Core Laboratory includes a sequence of procedures: sample preparation and homogenisation, DNA extraction, fragmentation and purification. Detailed protocols are available on protocols.io ( Denton *et al.*, 2023b). The icRhaMord1 sample was prepared for DNA extraction by weighing and dissecting it on dry ice ( Jay *et al.*, 2023). Tissue was homogenised using a PowerMasher II tissue disruptor ( Denton *et al.*, 2023a).

HMW DNA was extracted in the WSI Scientific Operations core using the Automated MagAttract v2 protocol ( Oatley *et al.*, 2023). The DNA was sheared into an average fragment size of 12–20 kb in a Megaruptor 3 system ( Bates *et al.*, 2023). Sheared DNA was purified by solid-phase reversible immobilisation, using AMPure PB beads to eliminate shorter fragments and concentrate the DNA ( Strickland *et al.*, 2023). The concentration of the sheared and purified DNA was assessed using a Nanodrop spectrophotometer and Qubit Fluorometer using the Qubit dsDNA High Sensitivity Assay kit. Fragment size distribution was evaluated by running the sample on the FemtoPulse system.

### Hi-C preparation

Tissue from the icRhaMord1 sample was processed at the WSI Scientific Operations core, using the Arima-HiC v2 kit. Tissue (stored at –80 °C) was fixed, and the DNA crosslinked using a TC buffer with 22% formaldehyde. After crosslinking, the tissue was homogenised using the Diagnocine Power Masher-II and BioMasher-II tubes and pestles. Following the kit manufacturer's instructions, crosslinked DNA was digested using a restriction enzyme master mix. The 5’-overhangs were then filled in and labelled with biotinylated nucleotides and proximally ligated. An overnight incubation was carried out for enzymes to digest remaining proteins and for crosslinks to reverse. A clean up was performed with SPRIselect beads prior to library preparation.

### Library preparation and sequencing

Library preparation and sequencing were performed at the WSI Scientific Operations core. Pacific Biosciences HiFi circular consensus DNA sequencing libraries were prepared using the PacBio Express Template Preparation Kit v2.0 (Pacific Biosciences, California, USA) as per the manufacturer's instructions. The kit includes the reagents required for removal of single-strand overhangs, DNA damage repair, end repair/A-tailing, adapter ligation, and nuclease treatment. Library preparation also included a library purification step using AMPure PB beads (Pacific Biosciences, California, USA) and size selection step to remove templates shorter than 3 kb using AMPure PB modified SPRI. DNA concentration was quantified using the Qubit Fluorometer v2.0 and Qubit HS Assay Kit and the final library fragment size analysis was carried out using the Agilent Femto Pulse Automated Pulsed Field CE Instrument and gDNA 165kb gDNA and 55kb BAC analysis kit. Samples were sequenced using the Sequel IIe system (Pacific Biosciences, California, USA). The concentration of the library loaded onto the Sequel IIe was in the range 40–135 pM. The SMRT link software, a PacBio web-based end-to-end workflow manager, was used to set-up and monitor the run, as well as perform primary and secondary analysis of the data upon completion.

For Hi-C library preparation, DNA was fragmented to a size of 400 to 600 bp using a Covaris E220 sonicator. The DNA was then enriched, barcoded, and amplified using the NEBNext Ultra II DNA Library Prep Kit following manufacturers’ instructions. The Hi-C sequencing was performed using paired-end sequencing with a read length of 150 bp on an Illumina NovaSeq 6000 instrument.

### Genome assembly, curation and evaluation

***Assembly***

The HiFi reads were first assembled using Hifiasm ( Cheng *et al.*, 2021) with the --primary option. Haplotypic duplications were identified and removed using purge_dups ( Guan *et al.*, 2020). The Hi-C reads were mapped to the primary contigs using bwa-mem2 ( Vasimuddin *et al.*, 2019). The contigs were further scaffolded using the provided Hi-C data ( Rao *et al.*, 2014) in YaHS ( Zhou *et al.*, 2023) using the --break option for handling potential misassemblies. The scaffolded assemblies were evaluated using Gfastats ( Formenti *et al.*, 2022), BUSCO ( Manni *et al.*, 2021) and MERQURY.FK ( Rhie *et al.*, 2020).

The mitochondrial genome was assembled using MitoHiFi ( Uliano-Silva *et al.*, 2023), which runs MitoFinder ( Allio *et al.*, 2020) and uses these annotations to select the final mitochondrial contig and to ensure the general quality of the sequence.

***Assembly curation***

The assembly was decontaminated using the Assembly Screen for Cobionts and Contaminants (ASCC) pipeline (article in preparation). Flat files and maps used in curation were generated in TreeVal ( Pointon *et al.*, 2023). Manual curation was primarily conducted using PretextView ( Harry, 2022), with additional insights provided by JBrowse2 ( Diesh *et al.*, 2023) and HiGlass ( Kerpedjiev *et al.*, 2018). Scaffolds were visually inspected and corrected as described by Howe *et al.* (2021). Any identified contamination, missed joins, and mis-joins were corrected, and duplicate sequences were tagged and removed. The curation process is documented at https://gitlab.com/wtsi-grit/rapid-curation (article in preparation).

***Assembly quality assessment***

The Merqury.FK tool ( Rhie *et al.*, 2020), run in a Singularity container ( Kurtzer *et al.*, 2017), was used to evaluate *k*-mer completeness and assembly quality for the primary and alternate haplotypes using the *k*-mer databases ( *k* = 31) that were computed prior to genome assembly. The analysis outputs included assembly QV scores and completeness statistics.

A Hi-C contact map was produced for the final version of the assembly. The Hi-C reads were aligned using bwa-mem2 ( Vasimuddin *et al.*, 2019) and the alignment files were combined using SAMtools ( Danecek *et al.*, 2021). The Hi-C alignments were converted into a contact map using BEDTools ( Quinlan & Hall, 2010) and the Cooler tool suite ( Abdennur & Mirny, 2020). The contact map is visualised in HiGlass ( Kerpedjiev *et al.*, 2018).

The blobtoolkit pipeline is a Nextflow port of the previous Snakemake Blobtoolkit pipeline ( Challis *et al.*, 2020). It aligns the PacBio reads in SAMtools and minimap2 ( Li, 2018) and generates coverage tracks for regions of fixed size. In parallel, it queries the GoaT database ( Challis *et al.*, 2023) to identify all matching BUSCO lineages to run BUSCO ( Manni *et al.*, 2021). For the three domain-level BUSCO lineages, the pipeline aligns the BUSCO genes to the UniProt Reference Proteomes database ( Bateman *et al.*, 2023) with DIAMOND blastp ( Buchfink *et al.*, 2021). The genome is also divided into chunks according to the density of the BUSCO genes from the closest taxonomic lineage, and each chunk is aligned to the UniProt Reference Proteomes database using DIAMOND blastx. Genome sequences without a hit are chunked using seqtk and aligned to the NT database with blastn ( Altschul *et al.*, 1990). The blobtools suite combines all these outputs into a blobdir for visualisation.

The blobtoolkit pipeline was developed using nf-core tooling ( Ewels *et al.*, 2020) and MultiQC ( Ewels *et al.*, 2016), relying on the Conda package manager, the Bioconda initiative ( Grüning *et al.*, 2018), the Biocontainers infrastructure ( da Veiga Leprevost *et al.*, 2017), as well as the Docker ( Merkel, 2014) and Singularity ( Kurtzer *et al.*, 2017) containerisation solutions.

Table 4 contains a list of relevant software tool versions and sources.

**Table 4.** Software tools: versions and sources.

Software tool	Version	Source
BEDTools	2.30.0	https://github.com/arq5x/bedtools2
BLAST	2.14.0	ftp://ftp.ncbi.nlm.nih.gov/blast/executables/blast+/
BlobToolKit	4.3.7	https://github.com/blobtoolkit/blobtoolkit
BUSCO	5.4.3 and 5.5.0	https://gitlab.com/ezlab/busco
bwa-mem2	2.2.1	https://github.com/bwa-mem2/bwa-mem2
Cooler	0.8.11	https://github.com/open2c/cooler
DIAMOND	2.1.8	https://github.com/bbuchfink/diamond
fasta_ windows	0.2.4	https://github.com/tolkit/fasta_windows
FastK	427104ea91c78c3b8b8b49f1a7d6bbeaa869ba1c	https://github.com/thegenemyers/FASTK
Gfastats	1.3.6	https://github.com/vgl-hub/gfastats
GoaT CLI	0.2.5	https://github.com/genomehubs/goat-cli
Hifiasm	0.19.8-r587	https://github.com/chhylp123/hifiasm
HiGlass	44086069ee7d4d3f6f3f0012569789ec138f42b84a a44357826c0b6753eb28de	https://github.com/higlass/higlass
Merqury.FK	d00d98157618f4e8d1a9190026b19b471055b22e	https://github.com/thegenemyers/MERQURY.FK
MitoHiFi	3	https://github.com/marcelauliano/MitoHiFi
MultiQC	1.14, 1.17, and 1.18	https://github.com/MultiQC/MultiQC
NCBI Datasets	15.12.0	https://github.com/ncbi/datasets
Nextflow	23.04.0-5857	https://github.com/nextflow-io/nextflow
PretextView	0.2.5	https://github.com/sanger-tol/PretextView
purge_ dups	1.2.5	https://github.com/dfguan/purge_dups
samtools	1.16.1, 1.17, and 1.18	https://github.com/samtools/samtools
sanger-tol/ ascc	-	https://github.com/sanger-tol/ascc
Seqtk	1.3	https://github.com/lh3/seqtk
Singularity	3.9.0	https://github.com/sylabs/singularity
TreeVal	1.0.0	https://github.com/sanger-tol/treeval
YaHS	1.2a.2	https://github.com/c-zhou/yahs

### Genome annotation

The Ensembl Genebuild annotation system ( Aken *et al.*, 2016) was used to generate annotation for the *Rhagium mordax* assembly (GCA_963680705.1) in Ensembl Rapid Release at the EBI. Annotation was created primarily through alignment of transcriptomic data to the genome, with gap filling via protein-to-genome alignments of a select set of proteins from UniProt ( UniProt Consortium, 2019).

### Wellcome Sanger Institute – Legal and Governance

The materials that have contributed to this genome note have been supplied by a Darwin Tree of Life Partner. The submission of materials by a Darwin Tree of Life Partner is subject to the **‘Darwin Tree of Life Project Sampling Code of Practice’**, which can be found in full on the Darwin Tree of Life website here. By agreeing with and signing up to the Sampling Code of Practice, the Darwin Tree of Life Partner agrees they will meet the legal and ethical requirements and standards set out within this document in respect of all samples acquired for, and supplied to, the Darwin Tree of Life Project.

Further, the Wellcome Sanger Institute employs a process whereby due diligence is carried out proportionate to the nature of the materials themselves, and the circumstances under which they have been/are to be collected and provided for use. The purpose of this is to address and mitigate any potential legal and/or ethical implications of receipt and use of the materials as part of the research project, and to ensure that in doing so we align with best practice wherever possible. The overarching areas of consideration are:

•     Ethical review of provenance and sourcing of the material

•     Legality of collection, transfer and use (national and international)

Each transfer of samples is further undertaken according to a Research Collaboration Agreement or Material Transfer Agreement entered into by the Darwin Tree of Life Partner, Genome Research Limited (operating as the Wellcome Sanger Institute), and in some circumstances other Darwin Tree of Life collaborators.

## Data Availability

European Nucleotide Archive: *Rhagium mordax*. Accession number PRJEB63422; https://identifiers.org/ena.embl/PRJEB63422. The genome sequence is released openly for reuse. The *Rhagium mordax* genome sequencing initiative is part of the Darwin Tree of Life (DToL) project. All raw sequence data and the assembly have been deposited in INSDC databases. Raw data and assembly accession identifiers are reported in Table 1 and Table 2.

## Author information

Members of the Natural History Museum Genome Acquisition Lab are listed here: https://doi.org/10.5281/zenodo.12159242.

Members of the Darwin Tree of Life Barcoding collective are listed here: https://doi.org/10.5281/zenodo.12158331.

Members of the Wellcome Sanger Institute Tree of Life Management, Samples and Laboratory team are listed here: https://doi.org/10.5281/zenodo.12162482.

Members of Wellcome Sanger Institute Scientific Operations: Sequencing Operations are listed here: https://doi.org/10.5281/zenodo.12165051.

Members of the Wellcome Sanger Institute Tree of Life Core Informatics team are listed here: https://doi.org/10.5281/zenodo.12160324.

Members of the Tree of Life Core Informatics collective are listed here: https://doi.org/10.5281/zenodo.12205391.

Members of the Darwin Tree of Life Consortium are listed here: https://doi.org/10.5281/zenodo.4783558.
