# Supplementary material for: The genome sequence of a longhorn beetle, Rhagium mordax (Degeer, 1775)
Source: Wellcome Open Res. 2025 Feb 7;10:51. [Version 1] doi: 10.12688/wellcomeopenres.23668.1 (PMC11909496; doi:10.12688/wellcomeopenres.23668.1)
Supplement: Peer review history

# Reviewer report: Reviewer response for version 1

**DOI:** 10.21956/wellcomeopenres.26111.r119398

**Published:** 2025-03-14

**Authors:** Jay K Goldberg

**Affiliations:** 1 Arizona State University School of Life Sciences (Ringgold ID: 69991), Tempe, Arizona, USA

This report summarizes yet another high-quality genome assembly from the team at DToL. This time they've sequenced a beetle (Rhagium mordax). The background section is longer and provides more natural history background of the species - something I appreciated after having previously reviewed reports with more minimal introductions.

 The methods are sound, thoroughly described, and represent the state-of-the-art in genome assembly. One minor complain is that some collection details are provided in the Background, but would be better placed with "Sample acquisition and DNA barcoding" in the Methods section. I appreciated Table 4, which clearly lists the software/versions used.

 Another minor issue: Figure 5 should have axis labels of some kind (position in bps and/or Chr designations).

Are sufficient details of methods and materials provided to allow replication by others?

Yes

Is the rationale for creating the dataset(s) clearly described?

Yes

Are the datasets clearly presented in a useable and accessible format?

Yes

Are the protocols appropriate and is the work technically sound?

Yes

Reviewer Expertise:

Entomology, genomics, plant-insect interactions

I confirm that I have read this submission and believe that I have an appropriate level of expertise to confirm that it is of an acceptable scientific standard.

# Reviewer report: Reviewer response for version 1

**DOI:** 10.21956/wellcomeopenres.26111.r119400

**Published:** 2025-03-11

**Authors:** Bin Zhang

**Affiliations:** 1 China-Australia Joint Institute of Agricultural and Environmental Health, Qingdao Agricultural University, Shenzhen, China

1.Although the manuscript provides detailed descriptions of sample collection, DNA extraction, sequencing, and assembly processes, some steps—such as Hi-C data processing and post-assembly manual curation—lack sufficient detail. The authors are encouraged to include more specific parameters and procedural details to enable better reproducibility by peers.

 2.The manuscript reports several metrics, including N50, BUSCO, QV, and k-mer completeness, which overall indicate high quality. However, for certain metrics (for example, the slightly lower-than-expected k-mer completeness), a more detailed discussion and explanation is recommended to clarify the relationships between these metrics and any influencing factors.

 3.Genome annotation was performed using the Ensembl Genebuild system, and the numbers of protein-coding and non-coding genes are reported. It is suggested that the authors further expand on functional gene annotation, such as providing information on the distribution of specific functional categories or analyses linking genes to known biological processes, to enhance the utility of the data.

 4.While the figures and tables are generally clear, some legends and accompanying descriptions are somewhat brief. The authors should consider providing more detailed explanations for key figures (e.g., BlobToolKit plots and Hi-C contact maps) to ensure that readers fully understand the significance of the presented data.

Are sufficient details of methods and materials provided to allow replication by others?

Yes

Is the rationale for creating the dataset(s) clearly described?

Yes

Are the datasets clearly presented in a useable and accessible format?

Yes

Are the protocols appropriate and is the work technically sound?

Yes

Reviewer Expertise:

Entomology, Bioinformatics

I confirm that I have read this submission and believe that I have an appropriate level of expertise to confirm that it is of an acceptable scientific standard.
